# Fedratinib combined with ropeginterferon alfa-2b in patients with myelofibrosis (FEDORA): study protocol for a multicentre, open-label, Bayesian phase II trial

**DOI:** 10.1186/s12885-024-13383-3

**Published:** 2025-01-10

**Authors:** Graham McIlroy, Charlotte Gaskell, Aimee Jackson, Emily Yafai, Rachel Tasker, Catherine Thomas, Sonia Fox, Rebecca Boucher, Fitsum Ghebretinsea, Claire Harrison, Adam J. Mead, Mary Frances McMullin

**Affiliations:** 1 Cancer Research UK Clinical Trials Unit (CRCTU), University of Birmingham, Edgbaston, Birmingham, B15 2TT UK; 2 Department of Clinical Haematology, Guy’s and St Thomas’ National Health Service (NHS) Foundation Trust, London, UK; 3 Medical Research Council (MRC) Molecular Haematology Unit, MRC Weatherall Institute of Molecular Medicine, NIHR, Biomedical Research Centre, University of Oxford, Oxford, UK; 4 Centre for Medical Education, Queen’s University Belfast, Belfast City Hospital, Lisburn Road, Belfast, UK

**Keywords:** Myelofibrosis, Phase II, Clinical trial, Fedratinib, Ropeginterferon alfa-2b, JAK2 inhibition, Bayesian design, Tolerability

## Abstract

**Background:**

Myelofibrosis (MF) is a clonal haematopoietic disease, with median overall survival for patients with primary MF only 6.5 years. The most frequent gene mutation found in patients is *JAK2*^V617F^, causing constitutive activation of the kinase and activation of downstream signalling. Fedratinib is an oral selective JAK2 inhibitor. It has shown activity in MF and is well-tolerated, but combination with other therapies is likely needed to achieve clonal remission. Combining a JAK2 inhibitor with an interferon may be synergistic, as haematopoietic cells are activated from quiescence (a typical kinase resistance mechanism) rendering them more sensitive to inhibition.

Ropeginterferon alfa-2b is a next generation pegylated interferon-α-2b with high tolerability and clinical activity in patients with MF, however, evidence of tolerability and activity in combination with fedratinib is lacking in this setting. The aim of the FEDORA trial is to assess tolerability, safety, and activity of fedratinib with ropeginterferon alfa-2b in patients with MF who require treatment to justify further investigation in a phase III trial.

**Methods:**

FEDORA is a single arm, multicentre, open-label, Bayesian phase II trial to assess tolerability, safety, and activity of fedratinib with ropeginterferon alfa-2b aiming to recruit 30 patients. Patients with *JAK2*^V617F^ positive primary or secondary MF, who are aged ≥ 18 years, have intermediate-1 with palpable splenomegaly of > 5cm, intermediate-2, or high-risk disease according to the Dynamic International Prognostic Scoring System (DIPSS), and who require treatment are eligible. The primary outcome is tolerability, whereby the combination is deemed intolerable in a patient if drug-related toxicities in the first four months of treatment lead to: either drug being discontinued; delays in treatment exceeding 28 consecutive days; or death. FEDORA uses a within-patient dose escalation regimen to ensure each patient reaches a personalised dose combination that is acceptable.

**Discussion:**

FEDORA is using a Bayesian trial design and aims to provide evidence of the tolerability, safety, and activity of combining fedratinib with ropeginterferon alfa-2b upon which the decision as to whether a phase III trial is warranted will be based.

**Trial registration:**

EudraCT number: 2021–004056-42.

ISRCTN: 88,102,629.

**Supplementary Information:**

The online version contains supplementary material available at 10.1186/s12885-024-13383-3.

## Background

Myelofibrosis (MF) is a clonal haematopoietic disease characterised by progressive bone marrow fibrosis, splenomegaly, extramedullary haematopoiesis, anaemia, and constitutional symptoms [1]. MF is predominantly a disease of older age (median 66 years) [2] that can arise de novo (primary MF) or as a complication of essential thrombocythaemia or polycythaemia vera (PV) (secondary MF). The median overall survival of patients with primary MF is 6.5 years [3]. Apart from allogeneic stem cell transplantation, which is only applicable for a minority of younger patients with MF, treatment strategies have historically been palliative and for the most part ineffective.

Genetically, MF is a heterogeneous disease. Mutations have been reported in a number of genes, the most frequent of which is a gain-of-function, recurrent mutation *JAK2*^V617F^, causing constitutive activation of the kinase and activation of downstream signalling via *JAK2* [4]. Furthermore, many patients show activation of the JAK2 signalling pathway in the absence of the *JAK2* mutation, likely caused by mutations indirectly activating the JAK2 pathway, e.g., *MPL*. Inhibitors of JAK2 (JAK2i) have subsequently been developed and integrated into treatment guidelines [5, 6].

Fedratinib is an oral selective JAK2i with higher inhibitory activity for JAK2 than other family members [7]. It has a long half-life (41 h) and is, therefore, suitable for once daily dosing. In an early phase investigation in patients with MF, single agent fedratinib was well-tolerated, with encouraging activity and a reduction in disease burden [8]. Later phase trials also demonstrated spleen volume and symptom responses [9–11]. Despite reports of Wernicke’s encephalopathy, this has been re-evaluated with more detailed follow-up data, and the risk of serious consequences mitigated by thiamine monitoring and replacement [12]. Fedratinib has since been approved by the US Food and Drug Administration for adult patients with intermediate-2 or high-risk MF [13] and by the European Medicines Agency and UK’s Medicines and Healthcare products Regulatory Agency in adult patients with MF.

Despite encouraging results, JAK2i are unlikely to be curative, as clonal markers of disease remain detectable in almost all patients, including those demonstrating a clinical response. Additionally, responses to JAK2 inhibition are highly heterogeneous, ranging from stable/progressive disease (primary resistance) in some to complete resolution of splenomegaly in others. Therefore, combination of JAK2 inhibition with other therapies may induce synergistic responses and increase the chance of achieving clonal remission.

Interferon-α (IFN-α) has been used in the treatment of MF for over 30 years with clinical, haematological, and morphological responses seen [14, 15]. The mode of action remains unclear with pro-apoptotic effects on myeloid progenitors [16], host immune enhancing effects and restoring immune surveillance [17], and having direct effects on the malignant *JAK2*-mutant clone [18] reported. Ropeginterferon alfa-2b is a next generation pegylated IFN-α-2b, that has high tolerability and a longer half-life (allowing two–weekly dosing in general and four-weekly dosing in the maintenance phase), compared with conventional pegylated-IFN-α preparations [19]. Ropeginterferon alfa-2b has been shown to induce significant haematological and molecular responses in PV [20]. Early evidence in patients with pre-fibrotic primary MF indicates ropeginterferon alfa-2b can also induce haematological responses and improve quality of life in this setting [21, 22].

Combining a JAK2i and an interferon may be more effective as a MF treatment as haematopoietic cells are activated from quiescence (a typical mechanism of resistance to tyrosine kinase inhibitors) [23], which may render them more sensitive to inhibition. Combinations of a JAK2i and IFN-α have indeed demonstrated dramatic reductions in splenomegaly, improvement in constitutional symptoms, and reduction in leucocytosis and thrombosis, with tolerable side effects in patients with primary and secondary MF [24–27]. Therefore, the aim of the FEDORA trial is to assess tolerability, safety, and activity of fedratinib with ropeginterferon alfa-2b in patients with MF who require treatment to justify further investigation in a larger phase III trial.

## Methods

### Trial design

**Fig. 1:**
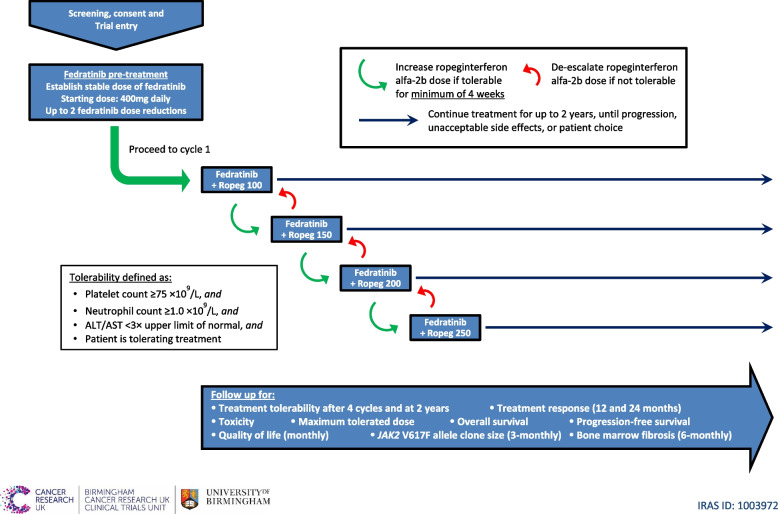
FEDORA trial schema. Overview of the FEDORA trial. Up to two dose reductions of fedratinib are permitted. A patient is defined as not tolerating treatment if they discontinue either fedratinib or ropeginterferon alfa-2b due to drug-related toxicity, due to delays in treatment exceeding 28 consecutive days due to drug-related toxicity, or if a treatment toxicity-related death is reported, within four months of starting combination therapy. ALT, alanine transferase; AST, aspartate aminotransferase; Ropeg, ropeginterferon alfa-2b

The FEDORA trial is a single-arm, open-label, phase II clinical trial in patients with primary or secondary MF that is intermediate-1 with palpable splenomegaly of > 5cm, intermediate-2, or high-risk according to DIPSS [28], and who require treatment. FEDORA will recruit 30 patients across 12 UK centres to establish whether the combination of fedratinib with ropeginterferon alfa-2b is tolerable, safe, and clinically active using a within-patient dose escalation regimen to ensure each patient reaches a personalised dose combination that is acceptable. A trial schema is presented in Fig. 1. The trial uses a Bayesian design to allow a judgement to be made on whether a phase III trial is warranted.

This trial aims to recruit patients over 18 months*,* with participants followed up monthly for two years.

A list of the FEDORA trial sites can be requested from the FEDORA Trial Office (FEDORA@trials.bham.ac.uk). The Standard Protocol Items: Recommendations for Intervention Trials (SPIRIT) checklist is provided as Appendix 1 [29]. The World Health Organization (WHO) Trial Registration Data Set is provided in Appendix 2.

### Patient and public involvement

A patient representative from the patient advisory group, MPN-Voice reviewed and refined the participant-facing documents. As a member of the Trial Steering Committee (TSC) they will assess study conduct throughout the duration of the trial, and will support dissemination of the study results via MPN-voice.

### Patient selection

Patients should be previously untreated with a JAK2 inhibitor or with an interferon alfa, and may be newly-diagnosed patients. Full eligibility criteria for FEDORA are listed in Table 1.

**Table 1:** Key patient eligibility criteria for the FEDORA trial

**Inclusion Criteria**
Age 18 or over at trial entry
Confirmed diagnosis of primary (overt) or secondary MF, according to WHO 2016 diagnostic criteria
*JAK2*^V617F^ positive, according to routine local assessment
Require treatment, as clinically determined by local investigator
Intermediate-1 with palpable splenomegaly > 5cm, intermediate-2 or high-risk according to DIPSS
Peripheral blood or bone marrow blasts < 10%
Adequate blood counts • Platelet count ≥ 75 × 10^9^/L • Neutrophil count ≥ 1.0 × 10^9^/L
Adequate organ function: • Creatinine clearance ≥ 30 mL/min (as estimated by the Cockcroft-Gault equation) • Serum bilirubin concentration < 1.5 × ULN • Serum alanine ALT *or* AST concentration < 3 × ULN • No worse than mild liver impairment (Child–Pugh class A is permitted)
ECOG performance score ≤ 2
Willing and able to comply with scheduled visits, treatment plan and other study procedures
Willing and able to provide written informed consent for the trial
**Exclusion Criteria**
Any previous treatment with a JAK2 inhibitor
Any previous treatment with interferon-α
Chemotherapy or biologic therapy within 2 weeks of commencing the trial treatment, or ongoing toxicity relating to prior therapy
Blood thiamine concentration below LLN • Before starting treatment, thiamine should be replenished
Active malignancy treated in the last 2 years, except: • Non-melanoma skin cancer • Carcinoma in situ of the cervix or breast • Incidental finding of prostate cancer (T1a or T1b)
Pre-existing thyroid disease unless controlled with conventional treatment
Uncontrolled diabetes
Autoimmune disease
Presence or history of severe psychiatric disorder, including severe depression, suicidal ideation, and suicide attempt
Severe cardiovascular disease, including uncontrolled hypertension, congestive heart failure (NYHA class ≥ 2), serious cardiac arrhythmia, significant coronary artery stenosis, unstable angina, or recent stroke of myocardial infarction that in the opinion of the Investigator makes the patient unsuitable for the trial
Previous organ transplantation receiving immunosuppression
Evidence of active HIV, hepatitis B, or hepatitis C infection, except: • HIV positive patients established on anti-retroviral treatment, with undetectable HIV RNA, after discussion with the patient’s HIV physician • Hepatitis B core antibody positive patients who are (i) surface antigen negative and (ii) hepatitis B DNA PCR negative, who take prophylaxis as per institutional guidelines
Predicted survival of less than 6 months
Allergy or hypersensitivity to fedratinib or ropeginterferon alfa-2b
Patients who are pregnant or breastfeeding (patients of childbearing potential^a^ must have a negative urine or serum pregnancy test prior to trial entry)
Patients and those with partners of childbearing potential^a^ not willing to use highly effective contraception during and for at least 1 month after discontinuation of therapy

*ALT* alanine transferase, *AST* aspartate aminotransferase, *DIPSS* Dynamic International Prognostic Scoring System, *ECOG* Eastern Cooperative Oncology Group, *LLN* lower limit of normal, *MF* myelofibrosis, *ULN* upper limit of normal

^a^Childbearing potential is defined as a pre-menopausal person capable of becoming pregnant. Patients will be considered post-menopausal if they have had no menses for 12 months without an alternative medical cause. A high follicle stimulating hormone (FSH) level in the postmenopausal range may be used to confirm a post-menopausal state in patients not using hormonal contraception or hormonal replacement therapy. However, in the absence of 12 months of amenorrhea, a single FSH measurement is insufficient

### Screening and consent

Potential patients are identified at haemato-oncology multidisciplinary team meetings at participating hospitals and/or identified on their first presentation with MF, for example through referral to haematology from primary care or on emergency admission to hospital. Patients will be given a FEDORA patient information sheet by the site investigator who will later take written informed consent, if agreed by the patient. Appendix 3 contains exemplar FEDORA trial patient information sheet, with Appendix 4 the informed consent form.

### Interventions

Table 2 provides an overview of a patient’s journey during the FEDORA trial.

**Table 2:** FEDORA trial schedule of events

Assessment		Screening (within 60 days of registration)	Fedratinib pre-treatment Up to 3 cycles		Combination treatment					End of treatment visit^b^	
					Cycles 1–2		Cycle 3	Cycles 4 +	Follow-up on treatment		
			Day 1	Day 15	Day 1	Day 15	Day 1	Day 1	(Month indicated)^a^		
Informed consent		x									Follow up 3-mothly after treatment for survival, progression and subsequent treatment
Demographic data and medical history		x									
Clinical & physical assessment (Height^c^, weight, ECOG status, blood pressure, pulse, oxygen sats, respiratory rate, physical examination, fundoscopy^c^)		x	x	x	x	x	x	x		x	
ECG		x									
Pregnancy test^d^		x	x		x		x	x		x	
Laboratory Tests	Haematology^e^	x	x	x	x	x	x	x		x	
	Blast count/percentage^f^	x	x	x	x	x	x	x		x	
	Blood film assessment	x							6, 12, 18, 24		
	Biochemistry^g^	x	x	x	x	x	x	x		x	
	Coagulation^h^	x	x		x		x		6, 9, 12, 15, 18, 21, 24		
	Thiamine (vitamin B1) testing	x	After every 3 months of fedratinib treatment								
	Virology^i^	x									
	*JAK2*^V617F^ assessment^j^	x									
Spleen assessment	Palpation	x	x		x		x	x			
	Ultrasound	x							6, 12, 18, 24		
Bone marrow aspirate and trephine		x							6, 12, 18, 24		
Peripheral blood and serum samples for research, including *JAK2*^V617F^ clone size		x			D1 C2 only		x		6, 9, 12, 15, 18, 21, 24		
DIPSS score		x									
MFSAF		x							3, 6, 9, 12, 15, 18, 21, 24		
Disease response assessment (IWG criteria)									6, 12, 18, 24		
WHO bleeding score		x	x		x		x	x			
Fedratinib administration			Daily throughout the trial								
Ropeginterferon alfa-2b administration					Day 1 and 15 of each cycle, starting from Cycle 1						
Thiamine supplementation			Daily, alongside fedratinib treatment								
Transfusion assessment		x	Monitored continuously								
Concomitant medication			Monitored continuously								
Adverse events		x^k^	Monitored continuously								

*DIPSS* dynamic international prognostic scoring system, *ECG* echocardiogram, *ECOG* Eastern Cooperative Oncology Group, *IWG* internal working group, *MFSAF* myelofibrosis symptom assessment form, *WHO* World Health Organization

^a^Within 14 days of time-points indicated

^b^To be completed 28 days after last dose of protocolised drug

^c^To be performed at screening. An eye examination should be performed immediately/as soon as possible for any patients experiencing a decrease or loss of vision during treatment

^d^For women of childbearing potential, to be performed within 14 days of first dose of study drug, at the start of each cycle of treatment and at the end of treatment visit

^e^Haematology to include: haemoglobin, platelets, white blood count (WBC), neutrophils, lymphocytes, and blast count/percentage. May be ± 3 days prior to the start of each cycle

^f^Blast count/percentage as recorded by routine analyser differential, or manual differential from blood film

^g^Biochemistry to include: albumin, bilirubin, alanine transferase/aspartate aminotransferase, alkaline phosphatase, calcium, creatinine, potassium, glomerular filtration rate, total protein, urea, amylase or lipase, sodium, triglycerides, and cholesterol. May be ± 3 days prior to the start of each cycle

^h^Coagulation to include prothrombin time (PT) *or* PT ratio *or* international normalised ratio (INR) and activated partial thromboplastin time (APTT) *or* APPT ratio

^i^Virology to include hepatitis B, hepatitis C, and HIV

^j^Can have been performed at any point prior to registration

^k^Serious adverse events collected from the point of consent, adverse events from the start of trial treatment

The expected doses of each drug are shown in Table 3. An initial pre-treatment stage of fedratinib monotherapy is followed by combination therapy with ropeginterferon alfa-2b from cycle 1. Consecutive 28-day cycles comprise oral fedratinib taken once daily continuously, and subcutaneous injections of ropeginterferon alfa-2b administered on days 1 and 15 of each cycle.

**Table 3:** Doses of fedratinib and ropeginterferon alfa-2b

Cycle number (each 28 days)	Fedratinib	Ropeginterferon alfa-2b
Pre-treatment	400 mg once daily	None
1	400 mg once daily	100 µg every two weeks
2	400 mg once daily	150 µg every two weeks
3	400 mg once daily	200 µg every two weeks
4	400 mg once daily	250 µg every two weeks
5–24	400 mg once daily	250 µg every two weeks

Patients will be treated for up to two years (24 cycles). On completion, patients are expected to revert to treatments routinely available as part of NHS care.

### Dose modifications, delays, and discontinuations

Patients should discontinue trial treatment in the following circumstances:

- Stopped by an investigator for clinical reasons not related to the study drug treatment.
- Any unforeseen event which, in the judgement of the investigator, makes further treatment inadvisable.
- Serious violation of the study protocol, including persistent patient non-attendance and persistent non-compliance.
- If the patient becomes pregnant or is a patient of child-bearing potential who fails to use adequate birth control.
- Serious adverse event (SAE) requiring permanent discontinuation of treatment.
- Development of unacceptable side effects.
- Confirmed disease progression (spleen size increase from baseline of > 5cm, increase in bone marrow fibrosis, or peripheral blood or bone marrow blasts ≥ 20%).
- At the request of the patient.

In the event of an overdose of either fedratinib or ropeginterferon alfa-2b, treatment should be paused, the patient closely monitored, and supportive measures provided as necessary. Any overdose with or without associated adverse effects (AEs) or SAEs is required to be reported within 24 h of first knowledge of the event to the Trial Office.

#### Fedratinib

During the pre-treatment stage, a tolerable dose of fedratinib must be established for 28 days (one cycle) before ropeginterferon alfa-2b can be started. Two dose reductions are permitted; to 300mg daily and then to 200 mg daily. Patients who require more than two dose reductions during fedratinib monotherapy should not continue to combination therapy and should be withdrawn from the trial.

The dose modifications for haematological and non-haematological toxicities, and treatment of emergent toxicities and management of Wernicke’s encephalopathy are shown in the Appendix 5. If the adverse reaction that results in a dose reduction is controlled, and the toxicity is resolved for at least 28 days, then the dose level may be re-escalated to one dose level higher per month up to the original dose level. Dose re-escalation is not recommended if the dose reduction was due to a grade 4 non-haematological toxicity, grade 3–4 alanine transferase, aspartate aminotransferase, or total bilirubin elevation, or re-occurrence of a grade 4 haematological toxicity.

Fedratinib should be reduced to 200mg once daily if concomitant treatment with strong CYP3A4 inhibitors cannot be avoided and patients carefully monitored. If the strong CYP3A4 inhibitor is discontinued, fedratinib should be increased to 300mg once daily during the first two weeks, and then 400mg once daily thereafter as tolerated.

#### Ropeginterferon alfa-2b

The starting dose of ropeginterferon alfa-2b is 100µg every two weeks, which is increased as permitted in Table 3. In case of grade ≥ 3 AE suspected to be related to the study drug, ropeginterferon alfa-2b should be paused and restarted only after recovery to grade 1 or baseline values. Ropeginterferon alfa-2b should then be restarted at one dose level lower (i.e., reduced by 50µg). The lowest permissible dose is 100µg.

Ropeginterferon alfa-2b therapy should be discontinued if, during treatment: renal function decreases significantly; patients develop symptoms indicative of a thyroid dysfunction and thyroid stimulating hormone levels cannot be controlled within the normal range; newly diagnosed diabetes mellitus cannot be controlled by medicinal products; any increase in liver enzyme levels is progressive and clinically significant; hepatic decompensation develops; if psychiatric symptoms worsen; and/or any patient develops new or worsening eye disorders. Investigators should refer to the current summary of product characteristics for additional guidance on toxicity management.

### Concomitant medication

Appendix 6 contains a list of prohibited concomitant medications, and those to be used with caution during FEDORA.

### Treatment compliance

The local trial pharmacist is responsible for maintaining and updating the drug accountability logs in the Pharmacy File throughout the study which will be used to monitor compliance. Unfinished bottles and unused pre-filled pens from discontinuing patients will be returned to the trial pharmacist who will count and document any unused medication. All investigational medical products may then be destroyed in accordance with local procedures and documented on the drug accountability log in the hospital Pharmacy File.

Patients are issued with a Patient Diary to complete each day, recording the time that each dose was taken, and whether any doses were missed. The diary also includes a section where the patient can record any relevant information such as side effects suffered or reasons for missed doses. The completed diary will be collected by the centre at each protocol scheduled visit and checked. Relevant information should be recorded on the case report form (CRF). The completed diary should be retained with the medical notes as source data.

### Trial outcomes

The primary outcome is tolerability of combination therapy. A patient is classified as not tolerating treatment if they discontinue either fedratinib or ropeginterferon alfa-2b due to drug-related toxicity, due to delays in treatment exceeding 28 consecutive days due to drug-related toxicity, or if a treatment toxicity-related death is reported, within four months of starting combination therapy.

The secondary outcomes are:

- Tolerability of combination therapy throughout the treatment course. A patient is classified as not tolerating treatment as per the definition for the primary outcome.
- Best overall response (complete plus partial response) assessed using International Working Group criteria (spleen size measure by palpation) [30] within 12 and 24 months from starting combination treatment. Patients who die or do not have a disease response recorded prior to the relevant time points will be classified as non-responders.
- The highest tolerated dose of ropeginterferon alfa-2b, in combination with fedratinib, achieved by each patient. To be tolerated, the dose must have been maintained for at least one complete cycle.
- Toxicity, defined to be any grade ≥ 3 AE, or a SAE of any grade. See the Adverse Events Reporting and Analysis section for details.
- Overall survival, defined as the time from starting combination therapy to date of death from any cause. Patients who are alive at the time of analysis or lost to follow-up will be censored at their date last seen.
- Progression-free survival, defined as the time from starting combination therapy to first event or death from any cause. An event is defined to be any of the following: an increase in bone marrow fibrosis, an increase in spleen size by > 5cm, or transformation to acute myeloid leukaemia. Patients who are alive and event-free at the time of analysis or lost to follow-up will be censored at their date last seen.
- Quality of life assessed using the myelofibrosis symptom assessment form (MFSAF) v4.0 [31] total symptom score at trial entry, and three-monthly during treatment.
- Bone marrow fibrosis, assessed using consensus definitions [32] at trial entry and six-monthly during treatment.

The *JAK2*^V617F^ clone size, measured at trial entry and three-monthly during treatment, is an exploratory outcome.

### Statistical analysis plan

#### Sample size

The sample size for FEDORA is 30 patients. For the fedratinib plus ropeginterferon alfa-2b combination treatment to be considered acceptable, 80% of patients would need to tolerate treatment. If 60% or fewer patients tolerated treatment, then the combination would be considered unacceptable. Based on this, if there is a high probability that the true tolerability rate is greater than 70% then the treatment would warrant further investigation. Employing a conjugate beta-binomial analysis, with a minimally informative Beta(1,1) prior, if 23 of the 30 patients tolerate the treatment combination we would be 76% sure that the true tolerability is greater than 70%, and therefore warrant further investigation (Appendix 7).

#### Interim and final analysis

No formal interim or pre-defined sub-group analyses are planned. Accumulating data and analyses will be monitored regularly by the TSC on a nine-monthly basis, or more frequently if required to monitor safety, recruitment, data quality, and activity. Final analysis of all outcome measures will be performed after all patients have completed a minimum of two years follow-up.

All analysis will be modified intention to treat (mITT) unless otherwise specified, with all patients who are recruited into the trial that receive at least one cycle of combination therapy included, irrespective of trial deviations and discontinuations. Patients who commence pre-treatment with fedratinib, but who do not then go on to receive concurrent ropeginterferon alfa-2b for any reason, or those who withdraw from the trial before the four-month primary outcome time-point for reasons unrelated to treatment toxicity or tolerability will be deemed unevaluable and replaced, and not included in the mITT analysis. Sensitivity analysis of outcomes will consider patient compliance with the treatment schedule and patients who do not receive at least one cycle of combination treatment.

#### Outcome analysis

For the primary outcome, the total number and proportion of patients tolerating four months of treatment will be reported alongside the associated 95% credible interval, using the total number of patients who started combination therapy as the denominator (including patients who failed to find any tolerable dose). Bayesian posterior probability plots will be presented alongside the probability that the true tolerability rate is greater than 70%.

Analyses of the secondary and exploratory outcomes will be descriptive in nature, with no hypothesis testing conducted.

- The total number and proportion of patients tolerating up to two years of combination therapy will be reported alongside the associated 95% credible interval, using the total number of patients who started combination therapy as the denominator.
- Best overall response rate will be presented as the number and proportion of patients achieving a response, overall and by response category, over the total number of patients receiving combination treatment.
- The number and proportion of patients will be reported against the highest tolerated ropeginterferon alfa-2b dose that each individual patient received, having followed dose escalation schedule.
- Toxicity will be presented as the number and proportion of patients experiencing one or more grade ≥ 3 AEs, or a SAE of any grade. This outcome will be analysed using the safety population.
- Overall and progression-free survival will be presented using Kaplan–Meier plots. The median time to event and survival estimates at one year will be presented with 95% confidence intervals where appropriate.
- The MFSAF v4.0 analyses will be presented as medians with interquartile ranges over time. Multi-level models may be used to compare changes in scores from baseline to 12 months if appropriate.
- Bone marrow fibrosis will be tabulated and presented graphically, with number and proportion of patients at each level reported. In addition to this, individual patient results will be plotted to show change over time at the patient level.
- The *JAK2*^V617F^ mutant allele frequency will be presented as medians and interquartile ranges over time, overall and split by highest dose of ropeginterferon alfa-2b tolerated and summarised using descriptive statistics. Individual patient plots will also be presented showing raw percentages.

### Adverse events reporting and analysis

The collection and reporting of AEs as measured by National Cancer Institute Common Terminology Criteria for Adverse Events (CTCAE), version 5.0 [33] will be in accordance with the Research Governance Framework for Health and Social Care and the requirements of the National Research Ethics Service. Definitions of different types of AEs are listed in Appendix 8. The reporting period for AEs will be between the date of commencement of protocol defined treatment until 28 days after the administration of the last protocolised drug. SAEs will be reported from the date of signing the Informed Consent Form to 28 days after the last dose of IMP. The investigator should assess the seriousness and causality (relatedness) of all AEs experienced by the patient with reference to the protocol. Abnormal laboratory findings should only be reported if the event results in the early discontinuation of trial treatment, a dose modification, or interruption. If a laboratory abnormality is one component of a diagnosis or syndrome, then only the diagnosis or syndrome should be recorded as an AE.

Pre-existing conditions should only be reported if the condition worsens by at least one CTCAE grade.

The following events should not be reported on an SAE Form:

- Hospitalisations for:
  - Protocol defined treatment.
  - Pre-planned elective procedures unless the condition worsens.
  - Treatment for progression of the patient’s cancer.
- Admissions or prolongations of hospitalisation due to social reasons.
- Progression or death because of the patient’s MF, as this information is captured elsewhere on the CRF.

The outcome of pregnancies of patients should be monitored and SAE data provided on congenital anomalies or birth defects.

Adverse events of special interest observed with fedratinib include:

- Encephalopathy, including Wernicke’s encephalopathy, or suspected cases of Wernicke’s encephalopathy with thiamine levels below normal range.

All cases of encephalopathy, of any grade, and whether or not the event is serious, should be reported using the SAE form.

### Collection and management of data and samples

FEDORA uses a secure online electronic remote data capture (eRDC) system to capture the CRF data. Access to the eRDC system is granted to individuals by the FEDORA Trial Office. Paper CRFs are also available as a backup. Data reported on each CRF should be consistent with the source data and any discrepancies explained. If information is not known, this must be clearly indicated on the CRF. All missing and ambiguous data will be queried. All sections are to be completed.

All trial records must be archived and securely retained for at least 20 years. No documents will be destroyed without prior approval from the sponsor, via the central FEDORA Trial Office. On-site monitoring will be carried out as required following a risk assessment and as documented in the Quality Management Plan. Any monitoring activities will be reported to the central Trial Office and any issues noted will be followed up to resolution. FEDORA is also centrally monitored, which may trigger additional on-site monitoring.

The CRCTU will hold the final trial dataset and will be responsible for the controlled sharing of anonymised clinical trial data with the wider research community to maximise potential patient benefit while protecting the privacy and confidentiality of trial participants. Data anonymised in compliance with the Information Commissioner’s Office requirements, using a procedure based on guidelines from the Medical Research Council Methodology Hubs, will be available for sharing with researchers outside of the trials team within 12 months of the primary publication.

Research samples collected during the trial will be stored at the Weatherall Institute for Molecular Medicine, and may be used for future ethically approved research if the patients who provided these samples have given their explicit consent.

### Trial organisation structure

The University of Birmingham will act as single sponsor: Research Governance & Integrity, Research Strategy and Services Division, Research Park, Birmingham, B15 2TT. Email: researchgovernance@contacts.bham.ac.uk. The trial is being conducted under the auspices of the CRCTU, University of Birmingham according to their local procedures. The trial management group (TMG) is responsible for the day-to-day running and management of the trial. Members of the TMG include the Lead Investigator, Co-Investigators, Clinical Coordinators, the Trial Management Team Leader (or delegate), the Trial Biostatistician, the Trial Coordinator, and the Monitor. The TMG meet three-monthly during recruitment and will then do so at least yearly.

An independent TSC has been set up to oversee the conduct of FEDORA. The TSC is chaired by an independent Chair with other members including a second independent clinician, an independent statistician, and one patient advocate. The TSC operates in accordance with a trial specific charter based upon the template created by the Damocles Group and, therefore, performs the role of a data monitoring committee. The TSC met seven months after the commencement of recruitment and have met at least nine-monthly since. The TSC advises on whether the accumulated trial data, together with the results from other relevant research, justifies the continuing recruitment of further patients. Additionally, the committee supervises the conduct of the trial, monitoring progress including recruitment, data completeness, losses to follow-up, and deviations from the protocol.

### Confidentiality

Confidential information collected during the trial will be stored in accordance with the General Data Protection Regulation 2018. As specified in the patient information sheet and with the patients consent, patients will be identified using only their date of birth and unique trial ID number. Authorised staff may have access to the records for quality assurance and audit purposes. The Trials Office maintains the confidentiality of all patients’ data and will not disclose information by which patients may be identified to any third party other than those directly involved in the treatment of the patient, and organisations for which the patient has given explicit consent for data transfer (e.g., laboratory staff).

### Dissemination of results and publication policy

Results will be submitted for publication in a peer reviewed journal. The manuscript will be prepared by the TMG, and authorship determined by mutual agreement in line with ICMJE criteria. A lay summary of the results will be published on the ISRCTN Results and CRCTU Trials websites. The trial protocol will be made available on the ISRCTN website.

Any secondary publications and presentations prepared by investigators will be reviewed by the TMG.

### Trial status

Recruitment for the trial opened in Sep-2022 and is expected to last until 31-Mar-2025.

## Discussion

### Choice of trial design

FEDORA has been designed as a single arm, phase II trial to assess the tolerability, safety, and activity of ropeginterferon alfa-2b in combination with fedratinib. Both fedratinib and ropeginterferon have been shown to be well-tolerated as single agents, however, there is less information on the tolerability and activity of these treatments in combination. As such, a single arm design was chosen to determine the tolerability of the combination treatment whilst assessing for evidence of activity within the secondary outcomes of best overall response and progression-free survival. If this trial produces a high probability that the true tolerability of the combination treatment is at least 70%, then further investigation in a larger trial would be warranted. A Bayesian approach was taken as opposed to a standard frequentist approach to improve the interpretability of the results obtained.

The within-patient dose escalation regimen has been designed to ensure each patient reaches a personalised dose combination that they are able to tolerate. Furthermore, the risk to the patient is reduced by requiring tolerability to single-agent fedratinib to be established first, followed by an escalation of ropeginterferon alfa-2b from a level far below that expected to cause significant side effects.

Evidence from FEDORA will be used to determine whether a phase III trial of fedratinib and ropeginterferon-alpha is warranted.

## Supplementary Information

Supplementary Material 1: Appendix 1: FEDORA SPIRIT checklist. A completed Standard Protocol Items: Recommendations for Intervention Trials (SPIRIT) checklist for the FEDORA protocol.

Supplementary Material 2: Appendix 2: The WHO trial registration dataset for FEDORA. The World Health Organization (WHO) trial registration data set for the FEDORA trial.

Supplementary Material 3: Appendix 3: FEDORA trial patient information sheet. Patient summary and information sheet for the FEDORA trial.

Supplementary Material 4: Appendix 4: FEDORA trial informed consent form. Exemplar informed consent form for the FEDORA trial.

Supplementary Material 5: Appendix 5: Dose modifications for fedratinib during FEDORA. The haematological and non-haematological toxicity guidance, as well as management of thiamine levels and Wernicke’s encephalopathy for use during FEDORA.

Supplementary Material 6: Appendix 6: Concomitant medications to avoid or use with caution during FEDORA. Drugs known or expected to interact with ropeginterferon alfa-2b or fedratinib.

Supplementary Material 7: Appendix 7: FEDORA Bayesian probability plots and operating characteristics. Posterior probability plots and operating characteristics for a range of possible scenarios within the FEDORA trial.

Supplementary Material 8: Appendix 8: Definitions of adverse events. Definitions of adverse events used during FEDORA.

## Data Availability

No datasets were generated or analysed during the current study.

## Abbreviations

CRF: Case report form
CTCAE: Common Terminology Criteria for Adverse Events
DIPSS: Dynamic International Prognostic Scoring System
eRDC: Electronic remote data capture
INF-α: Interferon-α
mITT: Modified intention to treat
MF: Myelofibrosis
MFSAF: Myelofibrosis symptom assessment form
PV: Polycythaemia vera
SAE: Serious adverse event
TMG: Trial management group
TSC: Trial steering committee
