# Supplementary material for: Fedratinib combined with ropeginterferon alfa-2b in patients with myelofibrosis (FEDORA): study protocol for a multicentre, open-label, Bayesian phase II trial
Source: BMC Cancer. 2025 Jan 10;25:56. doi: 10.1186/s12885-024-13383-3 (PMC11720754; doi:10.1186/s12885-024-13383-3)
Supplement: Supplementary file 2 — Supplementary Material 2: Appendix 2: The WHO trial registration dataset for FEDORA. The World Health Organization (WHO) trial registration data set for the FEDORA trial. [file 12885_2024_13383_MOESM2_ESM.docx]

# Supplementary Appendix 2

| **Data category** | **Information** |
| --- | --- |
| Primary registry and trial identifying number | ISRCTN: 88102629 |
| Date of registration in primary registry | 16-Jun-2022 |
| Secondary identifying numbers | EudraCT number: 2021-004056-42 |
| Source(s) of monetary or material support | Celgene  AOP Orphan  Cure Leukaemia |
| Primary sponsor | University of Birmingham |
| Secondary sponsor(s) | n/a |
| Contact for public queries | FEDORA Trial Coordinator ([FEDORA@trials.bham.ac.uk](mailto:FEDORA@trials.bham.ac.uk)) |
| Contact for scientific queries | FEDORA Trial Coordinator ([FEDORA@trials.bham.ac.uk](mailto:FEDORA@trials.bham.ac.uk)) |
| Public title | A study on the safety and effectiveness of fedratinib with ropeginterferon alfa-2b in patients with myelofibrosis |
| Scientific title | FEDORA: A phase II study to evaluate the tolerability, safety, and activity of fedratinib combined with ropeginterferon alfa-2b in patients with myelofibrosis |
| Countries of recruitment | UK |
| Health condition(s) or problem(s) studied | Myelofibrosis |
| Intervention(s) | Fedratinib with ropeginterferon alfa-2b |
| Key inclusion and exclusion criteria | Ages eligible for study: 18 years and over  Sexes eligible for study: both Accepts healthy volunteers: no |
|  | Inclusion criteria: *JAK2*^V617F^ positive primary or secondary myelofibrosis, intermediate-1 with palpable splenomegaly >5cm, intermediate-2, or high-risk according to DIPSS, requires treatment. |
|  | Exclusion criteria: Previous treatment with a JAK2 inhibitor or interferon-α, thiamine levels below lower limits of normal, other active malignancy, pregnancy or breastfeeding patients |
| Study type | Interventional |
|  | Allocation: open-label |
|  | Primary purpose: Tolerability |
|  | Phase II |
| Date of first enrolment | 08-Nov-2022 |
| Target sample size | 30 |
| Recruitment status | Open |
| Primary outcome(s) | Tolerability of combination therapy where a patient is classified as not tolerating treatment if they discontinue either fedratinib or ropeginterferon alfa-2b due to drug-related toxicity, due to delays in treatment exceeding 28 consecutive days due to drug-related toxicity, or if a treatment toxicity-related death is reported, within four months of starting combination therapy. |
| Key secondary outcome(s) | Tolerability of combination therapy throughout the treatment course. A patient is classified as not tolerating treatment as per the definition for the primary outcome.  Best overall response (complete plus partial response) assessed using International Working Group criteria (spleen size measure by palpation) within 12 and 24 months from starting combination treatment. Patients who die or don’t have a disease response recorded prior to the relevant time points will be classified as non-responders.  The highest tolerated dose of ropeginterferon alfa-2b, in combination with fedratinib, achieved by each patient. To be tolerated, the dose must have been maintained for at least one complete cycle.  Toxicity, defined to be any grade >3 adverse event, or a severe adverse event of any grade as measured by National Cancer Institute Common Terminology Criteria for Adverse Events, version 5.0.  Overall survival, defined as the time from starting combination therapy to date of death from any cause. Patients who are alive at the time of analysis or lost to follow-up will be censored at their date last seen.  Progression-free survival, defined as the time from starting combination therapy to first event or death from any cause. An event is defined to be any of the following: an increase in bone marrow fibrosis, an increase in spleen size by >5cm, or transformation to acute myeloid leukaemia. Patients who are alive and event-free at the time of analysis or lost to follow-up will be censored at their date last seen.  Quality of life assessed using the myelofibrosis symptom assessment form (MFSAF) v4.0 total symptom score at trial entry, and three-monthly during treatment.  Bone marrow fibrosis, assessed using consensus definitions at trial entry and six-monthly during treatment. |
