# Supplementary material for: Fedratinib combined with ropeginterferon alfa-2b in patients with myelofibrosis (FEDORA): study protocol for a multicentre, open-label, Bayesian phase II trial
Source: BMC Cancer. 2025 Jan 10;25:56. doi: 10.1186/s12885-024-13383-3 (PMC11720754; doi:10.1186/s12885-024-13383-3)
Supplement: Supplementary file 3 — Supplementary Material 3: Appendix 3: FEDORA trial patient information sheet. Patient summary and information sheet for the FEDORA trial. [file 12885_2024_13383_MOESM3_ESM.pdf]

Appendix 3: FEDORA trial patient information sheet

Print on hospital headed paper

PATIENT INFORMATION SHEET

FEDORA

FEDORA: A phase II study to evaluate the tolerability, safety and activity of fedratinib combined with ropeginterferon alfa-2b in patients with myelofibrosis

We would like to invite you to take part in a research study (also called a clinical trial) which is sponsored by the University of Birmingham. Joining the study is entirely up to you; before you decide we would like you to understand why the research is being done and what it would involve for you. Please take time to read this information carefully and discuss it with others if you wish.

Part 1 tells you the purpose of this study and what will happen to you if you take part, and part 2 gives you more detailed information about the conduct of the study. Do ask your research nurse or doctor if anything is unclear, or if you would like more information. Take time to decide whether or not you wish to take part.

If you choose not to take part, this will not affect the care you get from your own doctors. Thank you for reading this information sheet.

Contents

Part 1 — Page

What is the purpose of the FEDORA study? 2
Why have I been invited to take part? 3
How do I enter the study? 3
Do I have to take part? 3
What will happen to me if I take part? 4
What will happen if I don't want to carry on with the study? 9
What are the possible benefits of taking part? 9
What are the possible disadvantages and risks of taking part? 9

Part 2 — Page

Will I be paid to take part? 16
What to do if there are problems 16
What if new information becomes available? 17
Will my taking part in the trial be kept confidential? 17
What will happen to the results of the study 19
Will any genetic tests be done? 19
Who is organising and funding the study? 19
Who has reviewed the study 19
How have patients been involved? 20
Further information and contact details 20

Part 1 – Main Information Sheet

1 What is the purpose of the FEDORA study?

Myelofibrosis (MF) is a rare bone marrow cancer that causes scarring of the bone marrow. The scar tissue builds up inside your bone marrow leaving blood cells unable to develop properly. Symptoms of MF include anaemia (low red cell levels), weakness, tiredness and often an enlarged spleen. MF can develop without having had any other conditions; this is called primary myelofibrosis. Secondary myelofibrosis is where the condition develops in people who have other bone marrow disorders, such as polycythaemia vera or essential thrombocythaemia.

More than 55 in 100 people (more than 55%) with MF have a change in a gene called *JAK2*. The *JAK2* gene makes a protein that controls how many blood cells your body makes. JAK2 inhibitors are drugs that slow or stop the growth of cancer cells. They are also called cancer growth blockers. Although JAK2 inhibitors, such as ruxolitinib, and fedratinib, have shown promising results for some patients with MF, treatment with a JAK2 inhibitor does not usually cure the patient's disease.

Pegylated interferon is a type of immunotherapy that can be used to treat MF. Previous studies have shown that combining a JAK2 inhibitor with a pegylated interferon in patients with MF is safe to administer and may be more effective. This is thought to be because the interferon makes the stem cells that eventually develop into blood cells (haematopoietic cells) more sensitive to the JAK2 inhibitor.

The FEDORA study will use a selective JAK2 inhibitor called fedratinib, and a next generation pegylated interferon called ropeginterferon alfa-2b. Fedratinib has better activity against JAK2 than some other JAK2 inhibitors, and is given as an oral tablet. Ropeginterferon alfa-2b has been shown to be better tolerated than previous pegylated interferons and is self administered as an injection into the skin.

The aim of the FEDORA study is to gather more information about whether the combination of fedratinib and ropeginterferon alfa-2b is tolerated, and whether it provides a benefit to MF patients. The FEDORA study is for people with either primary or secondary myelofibrosis.

If you agree to take part you will begin your treatment with just fedratinib on its own (pre-treatment). This is to make sure your body is able to tolerate this treatment. After a month of pre-treatment, a low starting dose of ropeginterferon alfa-2b will be added to

your treatment regimen. This dose of ropeginterferon alfa-2b will be increased every month if you are doing well on the combination and your blood counts are stable or reduced if you are suffering with side effects. Once you have reached a dose combination that you are able to tolerate, you would remain on these doses for the duration of the study.

We will collect information about you and your disease, how it responds to treatment and any side effects you experience for 2 years after the start of your treatment.

2 Why have I been invited to take part?

Your doctor has invited you to consider taking part because you have been diagnosed with MF that requires treatment, and have the *JAK2* mutation. 30 patients from the UK will be participating in this study, and the study will take on new participants over an 18 month period.

We will collect information (data) on how each patient is doing for at least 2 years. Therefore, if you agree to participate, you will be part of the study for at least 2 years.

3 How do I enter the study?

The first step is to decide whether you want to take part in the study. Your doctor will describe the study and talk through this information sheet with you. This information sheet is yours to take away. If you choose to enter the FEDORA study, you will be invited to sign an Informed Consent Form to show that you understand what is involved when taking part in this study.

The original signed Informed Consent Form will be placed in your hospital notes. A copy will be given to you for your records, a copy will be sent to the FEDORA Trial Office, and a copy will be sent to the Weatherall Institute for Molecular Medicine (WIMM) at Oxford University where the samples you provide as part of this study will be analysed (please see the section “What samples will be collected?” for more information).

4 Do I have to take part?

No, participation in this study is entirely voluntary. If you consent to participate, you are still free to withdraw from the study at any time without giving a reason. If you decide not to take part, your treatment and standard of care will not be affected in any way and your doctor will discuss your treatment options with you. For more information see the section “What will happen if I don’t want to carry on with the study?”

5 What will happen to me if I take part?

Consent and Screening

If you decide to take part in this study, we will ask you to give your written informed consent to take part and the following tests will be performed to make sure you are suitable for the study. You would have most of these tests as part of your routine care, whether you take part in the study or not.

- A medical history, including medicines you currently take, and those you have taken in the past

- A physical exam including an eye exam, vital signs assessment and ECG (electrocardiogram) to measure your weight, height, temperature, pulse, heart rhythm and performance status (assessment of how your disease affects your daily living abilities) to assess your well-being before entering the study.

- A pregnancy test (if you are a female of child bearing potential)

- Blood tests – we will collect information about the results of several different blood tests as part of this study. It is likely that you have already had most of these done. However, if any extra blood tests are needed, your nurse will let you know and will arrange this.

  We will record information about the following blood tests:

  - Blood cell counts
  - Assessment of kidney and liver function
  - Assessment of the ability of your blood to clot
  - Measurement of the level of thiamine (vitamin B1) in your blood
  - Whether you have the *JAK2* mutation
  - Test to look for any viruses in your blood (such as Hepatitis and HIV)

    These tests are performed to check that you are well enough to begin study treatment.

  - A blood and serum sample will also be sent to the lab at Oxford University if you agree to this. More information about this is provided in the "What samples will be collected?" section.

- Spleen assessment. This will be performed by ultrasound and by your doctor pressing on your abdomen (palpation) to check your spleen
- Bone marrow aspirate and trephine for disease assessment (a sample of the aspirate and trephine will also be sent to the lab at Oxford University). More information about this is provided in the "What samples will be collected?" section.
- Dynamic international prognostic scoring system (DIPSS) score, which is a checklist your doctor will complete, and Myelofibrosis symptom assessment form (MFSAF), which is a questionnaire for you to complete to assess your symptoms.
- WHO bleeding score, to assess any bleeding due to your cancer
- Transfusion assessment, to determine your current need for a blood transfusion

You will need to have most of the tests described here as part of your treatment, whether or not you decide to enter this study. The extra tests that you will have as a result of entering this study are the measurement of blood thiamine levels, the eye examination, the DIPSS score and MFSAF.

Treatment

If you decide to take part in this study, you will be given a drug called fedratinib. This is given as oral capsules that should be swallowed whole with water, around the same time each day. You can take the capsules with or without food. For the first 28 days of the study, you will take fedratinib only (fedratinib pre-treatment), and the dose will be lowered if you feel too unwell. If you are able to tolerate the treatment, the dose will be kept at the same level for 28 days. Once you have completed 28 consecutive days of pre-treatment with fedratinib, you will start combination therapy.

For the combination therapy you will continue taking fedratinib each day, but will also be given ropeginterferon alfa-2b, to inject subcutaneously (into your skin) once every 2 weeks. Ropeginterferon alfa-2b is provided as a pre-filled pen for self-injection. Your doctor or research nurse will show you how to use the pre-filled pen to make sure you are confident to administer the treatment yourself. The dose of ropeginterferon alfa-2b will increase every cycle (every month) up to a maximum dose, as long as you are tolerating the therapy. If you are feeling too unwell, you doctor will decrease the dose given to you to find the most suitable amount of ropeginterferon alfa-2b for you.

You will also take thiamine (vitamin B1) supplements throughout your treatment on this study, as fedratinib is thought to lower the levels of thiamine in the body.

You can receive up to 2 years of treatment (24 cycles) while on the FEDORA study. Following this, your doctor will discuss what treatment you can continue with, including options that are available from the NHS at that time.

Assessments

The table below gives a description of the assessments required for this study. You would have most of these assessments as part of your routine care, whether or not you decide to enter this study. The extra tests that you will have as a result of entering this study are the measurement of blood thiamine levels and MFSAF. We will try to perform these during hospital visits which were already planned as part of your treatment.

Time point in the cycle	Tests or Procedures	Amount of time taken
Day 1 of each cycle of fedratinib pre-treatment	• Full physical examination, vital signs and weight<br>• Blood tests<br>• Assessment of your spleen by palpation<br>• WHO bleeding score<br>• Pregnancy test if you are a female of child-bearing potential	15 minutes<br>10 minutes<br>5 minutes<br>5 minutes<br>5 minutes
Day 15 of each cycle of fedratinib pre-treatment	• Full physical examination, vital signs and weight<br>• Blood tests	15 minutes<br>10 minutes
Day 1 and 15 of cycles 1 and 2 of combination therapy	• Full physical examination, vital signs and weight<br>• Blood tests<br>• Assessment of your spleen by palpation (day 1 only)<br>• Blood and serum samples collected for research (day 1 of cycle 2 only)<br>• WHO bleeding score (day 1 only)	15 minutes<br>10 minutes<br>5 minutes<br>10 minutes<br>5 minutes
Day 1 of subsequent cycles of combination therapy	• Full physical examination, vital signs and weight<br>• Blood tests<br>• Assessment of your spleen by palpation<br>• Blood and serum samples collected for research (cycle 3 only)<br>• WHO bleeding score (day 1 only)<br>• Pregnancy test if you are a female of child-bearing potential	15 minutes<br>10 minutes<br>5 minutes<br>10 minutes<br>10 minutes<br>5 minutes
	• Blood coagulation test (every 3 months)<br>• MF-SAF questionnaire (every 3 months)	5 minutes<br>5 minutes

Follow up during treatment	• Thiamine (vitamin B1) testing (every 3 months while taking fedratinib or if clinically indicated)<br>• Ultra sound (every 6 months)<br>• Bone marrow and trephine (every 6 months)<br>• Blood and serum samples collected for research (every 3 months)	5 minutes<br>30 minutes<br>30 minutes<br>10 minutes
**End of Treatment Visit – 28 days after final treatment**		
• Blood tests (including blood cell count) and physical examination and vital signs<br>• Pregnancy test if you are a female of child-bearing potential		20 minutes<br>10 minutes

If you stop trial treatment before the study ends, information concerning your disease status and the treatments you are receiving for your MF will be collected every 3 months. You do not need to attend any appointments or have any tests for this data to be collected.

<u>What samples will be collected?</u>

It is important to understand how different patients respond to this new treatment, and therefore, it is an integral part of the study to request blood and bone marrow samples for research purposes.

Bone marrow samples

Having bone marrow tests is an essential part of monitoring your response to treatment, and you will need to have these whether you enter this study or not.

There are two types of bone marrow tests: bone marrow aspirate and bone marrow trephine.
- A bone marrow aspirate is the removal of a small sample of liquid bone marrow.
- A bone marrow trephine is the removal of a tiny core of bone marrow which is about the thickness of a pencil lead and about 1-2cm (half an inch to an inch) long.

Whether or not you enter this study, you will need to have both types of bone marrow sample taken before starting treatment. You will also need to have follow-up bone marrow tests every 6 months during treatment, to monitor your MF, even if you do not take part in this study.

For this study, bone marrow aspirate and trephine samples will be analysed by your local hospital. A small drop of bone marrow and a thin slice of the bone marrow

trephine will be put onto separate glass slides; both of these samples will be looked at locally by your own study doctor or somebody in their team. A sample of aspirate (20mls or about 4 teaspoons) and of trephine will also be sent to the WIMM for further research into the genetics of MF.

Blood samples

Blood samples will be taken at the time points specified in the treatment schedule above. These will be taken whether you enter the study or not to monitor your disease and response to treatment. An additional 40mls of blood (approximately 8 teaspoons) and 20mls of serum (a separate blood test, about 4 teaspoons) will also be collected at the following time points to send to WIMM for further research; during screening, on day 1 of combination treatment cycles 2 and 3, and 3-monthly whilst on treatment from month 6. These samples will be taken at the same time as your routine blood test to avoid the discomfort of multiple blood draws.

What will happen to the samples that I give?

Samples of your blood and bone marrow will be stored at WIMM, where researchers will study how different patients respond to the treatment used during this study. This study involves analysis of blood cells, including their genetic material (DNA and RNA), with data from this securely stored and anonymised. It is expected that the FEDORA-specific research will be performed at WIMM, however, it is possible that other institutions may be asked to perform some or all of the FEDORA-specific research if it is not possible for this to be completed at WIMM. This may involve your samples being sent to research groups with appropriate ethical approval in place. These research groups may be outside Oxford and the European Economic Area (EEA), and may include academic and commercial collaborators. All samples will be sent without any identifiable information so that your identity is always protected.

After this research has been completed, samples may be stored at WIMM or transferred to a Human Tissue Authority approved biorepository (a place that collects, processes, stores, and distributes tissue samples to support future scientific investigation) for future research. Donation of your samples for future research is optional, and you can still enter the FEDORA study without agreeing to this.

Any research carried out on the samples will need to have ethical approval. Any samples stored will be anonymous and will not be able to be linked with you personally. If you consent to your samples being stored for future ethically approved research, there is the possibility that samples could be distributed to other laboratories during or after the

study has closed. In addition you will be asked if your samples can be used for other research that involves animals, this research will also be ethically approved. In addition, anonymised clinical data may be sent with your samples to allow researchers to analyse your samples in the most effective way.

6 What will happen if I don't want to carry on with the study?

You are free to withdraw from this study at any time, you do not have to give a reason and your future care will not be affected. If you decide to withdraw from the study treatment your doctor may ask you to return to hospital for follow up assessments for safety reasons. Your doctor will then discuss your further treatment options with you.

If you choose to withdraw from the study, we would still retain and analyse any information and samples we have collected up until withdrawal. If you decide to withdraw from the treatment only, you can decide whether to allow your hospital to continue to send information about your progress to the Trial Office. You will also be asked to return any unused medication, if applicable.

7 What are the possible benefits of taking part?

There is no guaranteed benefit to taking part in this study because we do not yet know whether this combination of treatments is better than the standard treatment. It is possible that the new treatment is not as good as the standard treatment. Equally, it is possible that the standard treatment is not as good which is why this study is being done. The careful monitoring you will receive if you take part in this study is a safeguard against this risk. The information gained from this study will help improve treatment for other people with MF in the future.

8 What are the possible disadvantages and risks of taking part?

What are the risks of the bone marrow aspirates and biopsies?

You will need to have bone marrow aspirate and trephine procedures performed whether or not you enter this study. Both of these processes may be painful but you will be given a local anaesthetic to numb the area before the tests are performed. However, it may be a little uncomfortable afterwards. The bone marrow aspirate takes approximately 5-10 minutes and if a trephine is also taken this will take an extra few minutes. You will normally have to lie down afterwards for half an hour. Please tell your doctor if you have any pain and you will be given painkillers.

The area will be covered with either a sticky plaster or a gauze pad. There may be a small amount of bleeding which is perfectly normal, but the doctor or nurse will make sure this has stopped before you go home.

If the site does start to bleed again, press on the area with a clean cloth or handkerchief. By pressing on the area, this will help your blood to clot and the bleeding to stop. The possible side effects associated with a bone marrow trephine include pain, bleeding, bruising and infection, as well as a reaction to the numbing agent.

<u>What are the side effects of treatment?</u>

It is possible that you will experience side effects from your treatment whether you take part in this study or not. Treatment for MF affects all healthy cells within the bone marrow as well as the cancer cells. This means that you might not be able to produce normal numbers of red blood cells, and you might need blood transfusions. If you are not able to produce enough platelets in your blood, you could be at risk of bleeding, so treatment might need to be reduced or you might need a platelet transfusion. More importantly the white blood cell count can fall, meaning that you could be at high risk of serious infections. Your doctor will instruct you about the steps to be taken if you develop a high temperature. If you do develop a temperature this can quickly turn into a life-threatening infection without prompt treatment so it is important that you receive medical attention and antibiotics straight away. Both fedratinib and Ropeginterferon alfa-2b are known to lower patients' blood counts.

You should tell your study doctor or medical team about any side effects that you have, even if you do not think they are connected to the drugs. Your doctor may be able to give you medications to help treat the side effects and prevent them from becoming worse. All side effects will be monitored closely to minimise any risks to you. Your study doctor may also choose to stop or delay treatment for a short time or reduce the dose to allow you to recover from any side effects.

Side effects associated with fedratinib

The side effects seen in patients taking fedratinib are listed below.

Side effects that have been seen in at least 1 in 10 participants (very common) include:

- decreased blood cells (red blood cells, platelets and white blood cells, described above)
- urinary tract infections
- bleeding
- headache
- loose or watery stools (diarrhoea)
- constipation
- nausea
- vomiting
- muscle spasms
- tiredness (fatigue)
- changes in blood test results (increased liver enzyme, creatinine, amylase and lipase levels. These may be signs of liver, kidney or pancreas problems

Side effects that have been seen in at least 1 in 100 participants (common) include

- Wernicke's encephalopathy (see below)
- dizziness
- hypertension (high blood pressure)
- indigestion (dyspepsia)
- bone pain
- pain in the extremities (neck, wrists, legs and arms)
- pain when urinating (dysuria)
- weight gain

Some people taking fedratinib have developed low thiamine (vitamin B1) levels, which can result in Wernicke's encephalopathy. This is a condition that affects your brain and leads to dizziness, blurred vision, memory loss and confusion. It is treated with thiamine, but if left untreated can lead to permanent brain damage. Your thiamine levels will be checked before and during your treatment, but if you experience any change in mental status, confusion or loss of memory you should let your doctor know immediately so

they can check your thiamine levels and provide treatment if needed. To reduce this risk, you will also take a thiamine tablet every day whilst on treatment.

Side effects associated with Ropeginterferon alfa-2b

Side effects that have been seen in at least 1 in 10 participants (very common) include:

- decreased blood cells (described above)
- joint and muscle pain
- flu-like symptoms
- tiredness (fatigue)

Side effects that have been seen in at least 1 in 100 participants (common) include:

- infections (respiratory tract infections and fungal skin infections)
- runny nose, sneezing and stuffiness
- changes in thyroid activity (under or overactive thyroid), or swelling of the thyroid
- decreased appetite
- depression and anxiety
- aggression, altered mood or mood swings
- sleep disorder
- headache, migraine or aura (migraine symptoms without a headache)
- dizziness
- dry eyes and mouth
- irregular heartbeat
- narrowing of small blood vessels
- shortness of breath
- diarrhoea
- constipation
- abdominal pain
- skin rash and dry skin
- hair loss
- arthritis
- pain in extremities, muscles and bone
- muscle spasms

- fever and chills
- injection site rash

Side effects that have been seen in at least 1 in 1000 participants (uncommon) include:

- sarcoidosis (small patches of red swollen tissue, often in the skin or lungs)
- Basedow’s disease (a disorder causing an over-active thyroid)
- diabetes
- severe depression
- nervousness and irritability
- hallucinations
- neuropathy (loss of sensation, pins and needles, numbness or pain)
- mental impairment
- tremor
- visual impairment
- deafness, tinnitus and vertigo
- cardiovascular disorders
- Raynaud's phenomenon (decreased blood flow to the fingers)
- Hypertension (high blood pressure)
- Haematoma (severe bruising)
- Hot flushes / flushing
- Cough and throat irritation
- Nosebleeds
- Gastrointestinal disorders (Indigestion, hernia, flatulence, frequent bowel movements)
- Difficulty swallowing
- Bleeding gums
- Liver disease
- Skin sensitivity to light
- Nail distortion and discolouration
- Muscle weakness
- Neck and groin pain
- Bladder infections, dysuria (pain when urinating) and urinary urgency
- Erectile dysfunction and blood in semen

If you experience any changes in your vision, please contact your doctor for an eye examination. You may need further eye examinations if you develop any new eye problems or if your vision worsens.

All treatments

Allergic reactions

Sometimes people have allergic reactions to drugs. Serious allergic reactions can be life-threatening. If you have an allergic reaction to one of these drugs, you might develop a rash, difficulty breathing, wheezing when you breathe, sudden low blood pressure with light-headedness, swelling around the mouth, throat or eyes, a racing heartbeat, and/or sweating. Before starting the study drug, you must tell your Study Doctor about any drug allergies. You should tell the Study Doctor right away if you have any allergy symptoms listed above.

Fatigue

If you are affected by fatigue, caution must be exercised when driving or using heavy machinery. Do not carry out these activities if you feel it is not safe to do so.

Harm to the unborn child

Information for Women

All of the drugs in this study may cause harm to an unborn child if administered during pregnancy. There is little or no information of the effects on the child when breast feeding during treatment with any of the drugs in this study.

You cannot take part in this study if you are pregnant, breast-feeding, planning to become pregnant or to do an egg donation while receiving study medication until 6 months after the last study drug administration. If you are a female who can become pregnant, you will be asked to take a pregnancy test prior to starting study drug treatment.

If you decide to take part in this study, you must agree to use two forms of effective contraception without interruption, including one barrier method (see box below) during the trial treatment and for 6 months after the last study drug administration.

If you become pregnant while receiving study medication or within 6 months after the last study drug administration, you must tell your doctor right away and any study medication you are taking will be discontinued. Your doctor will explain how to safeguard your health and the health of your baby. If you agree, we will collect Information related to the progress of your pregnancy and its outcome that is relevant to the study. This may include information related to your health, the date of conception, the course and outcome of your pregnancy and any medical treatments that you receive.

Information for Men

There is currently no information on the effects of the treatments in this study. If you were to father a child, the treatments you will receive as part of this study might be harmful to the unborn child.

If your partner might become pregnant, you/your partner must agree to use two effective forms of contraception, including one barrier method (see box below) during the trial treatment and for 6 months after the last study drug administration. Your doctor will talk to you about potential sperm donation before you start treatment and you should not be involved in sperm donation during this time. If your partner becomes pregnant during the course of the study, we would ask you to tell your study doctor immediately and your doctor will ask you and your partner for permission to collect information about the pregnancy and the child. Your doctor will explain to you and to your partner, how to safeguard your partner's health and the health of the baby.

The acceptable methods of effective contraception are: combined hormonal contraception or progestogen-only hormonal contraception associated with inhibition of ovulation, intrauterine device (IUD), intrauterine hormone-releasing system (IUS), bilateral tubal occlusion (a surgical procedure that involves blocking the fallopian tubes), a vasectomised partner, or sexual abstinence.

Barrier methods include: condom, diaphragm, cervical cap and contraceptive sponges with spermicide.

Please speak to your study doctor who will be able to provide appropriate contraception advice.

What food/medications (additional medication) should I avoid?

It is very important that you tell the study doctor about all medications, supplements, or herbal medicine that you are taking now and during the study. Even herbal medicines and other alternative treatments can interact with the treatments used in this study and the medicines you will be given to prevent infections and reduce side-effects, and these interactions could be dangerous.

Part 2 – Additional Information

1 Will I be paid to take part?

You will not receive any money for taking part in this study and unfortunately travel expenses cannot be reimbursed by the study organisers. However, some hospitals may offer travel expenses if you take part in the study and other transport services **may** be available. Please discuss access to these with your medical team.

2 What to do if there are problems

The main concern of everyone involved with this study is that your treatment is as safe, effective and tolerable as possible. If you have a concern about any aspect of this study, you should ask to speak to your doctor or study nurse who will do their best to answer your questions.

Complaints

If you remain unhappy and wish to complain formally, you can do this through the NHS Complaints Procedure. Details can be obtained from your hospital. Additionally, the contact information for your local Patient Advice and Liaison Service (PALS) or equivalent is at the end of this information sheet.

If you are harmed

If you are harmed by taking part in this study due to someone's negligence, then you may have grounds for a legal action for compensation but you may have to pay your legal costs. NHS Trusts have a duty of care to participants whether or not the participant is taking part in a clinical trial and normal National Health Service complaints mechanisms will still be available to you. If you have private medical insurance, you should tell your insurer that you are taking part in research. They will let you know if it affects your policy.

3 What if relevant new information becomes available?

If we get new information about the treatment being studied, your doctor will tell you and discuss whether you should continue in the study. If you decide not to carry on, your doctor will make arrangements for your care to continue. If you decide to continue in the study your doctor may ask you to sign an updated Informed Consent Form. If new information becomes available your doctor might consider that you should withdraw from the study. Your doctor will explain the reasons and arrange for your care to continue. If the study is stopped for any other reason, we will tell you and arrange your continuing care so you receive the best care available.

4 Will my taking part in the study be kept confidential?

All information collected about you for this study will be subject to the General Data Protection Regulation and Data Protection Act 2018 and will be kept strictly confidential.

All information collected by the Sponsor will be securely stored at the Trial Office at the Cancer Research UK Clinical Trials Unit, University of Birmingham on paper and electronically and will only be accessible by authorised personnel associated with the trial. The only people in the University of Birmingham who will have access to information that identifies you will be people who manage the study or audit the data collection process. When you are entered into the study we will collect your date of birth and initials. You will be given a unique study number and in routine communication between your hospital and the Trial Laboratory and Trial Office, you will only be identified by this study number. A copy of your signed consent form will be posted to the Trial Office to ensure that the correct consenting procedure has been carried out.

The NHS will use your name and contact details to contact you about the research study, and make sure that relevant information about the study is recorded for your care, and to oversee the quality of the trial.

Samples taken for research purposes and sent to the WIMM at Oxford University will be identified by your unique study number, date of birth and initials. This information is the minimum needed to make sure that your samples can be identified as yours.

In addition anonymised data (i.e. with patients initials removed) from the trial may be provided to other 3rd parties (e.g. pharmaceutical companies or other academic institutions) for research, safety monitoring or licensing purposes. This includes sending data to the manufacturers of the drugs (Bristol Myers Squibb and AOP Orphan

Pharmaceuticals) for safety monitoring purposes. They have the same duty of confidentiality to you as other personnel. These organisations could be within Europe, or outside Europe where the data protection laws may be different. Data sent abroad will not allow you as an individual to be identified.

By taking part in the study, you will be agreeing to allow research staff from the Trial Office at the University of Birmingham to look at the study records, including your medical records that are relevant to this study. It may be necessary to allow authorised personnel from government regulatory agencies (e.g. Medicines and Healthcare products Regulatory Agency (MHRA)), the Sponsor and/or NHS bodies to have access to your medical and research records. This is to ensure that the study is being conducted to the highest possible standards.

From time to time we may be asked to share the trial information (data) we have collected with researchers running other studies in this organisation and in other organisations so that they can perform analysis on the data to answer other important questions about MF. These organisations may be universities, NHS organisations or companies involved in health research and may be in this country or abroad. Any such request is carefully considered by the study researchers and will only be granted if the necessary procedures and approvals are in place. This information will not identify you and will not be combined with other information in a way that could identify you. The information will only be used for the purpose of health research, and cannot be used to contact you or to affect your care. It will not be used to make decisions about future services available to you, such as insurance. Under no circumstances will you be identified in any way in any report, presentation or publication arising from this or any other study.

You can withdraw your consent to our processing of your data at any time. Under the provisions of the General Data Protection Regulation (GDPR) 2018, you have the right to know what information the Trial Office has recorded about you. If you wish to view this information, or find more about how we use this information, please contact Legal Services at the address below or email dataprotection@contacts.bham.ac.uk.

Legal Services
University of Birmingham
Edgbaston
Birmingham, B15 2TT

Involvement of the General Practitioner (GP) /Family Practitioner

It is important that your GP is kept up to date with any treatment you are receiving. Your GP will be informed that you are taking part in this research study and they will be sent a copy of this information sheet.

5 What will happen to the results of the study?

When the study is complete the results will be published in a medical journal but no individual patients will be identified. If you would like to have a copy of the published results, please ask your study doctor or nurse.

6 Will any genetic tests be done?

Yes, please refer to the "Why have I been invited to take part?" section. During screening, your local hospital will test your samples for *JAK2* mutations. Your samples will also to be sent to the WIMM, where researchers will monitor the number of copies of *JAK2* in your samples to understand how you are responding to treatment. These tests only look for genetic changes which occurred during your life-time. They do not look for inherited genetic problems and so these results will not have consequences for your family members.

7 Who is organising and funding the study?

This research is being funded by Bristol Myers Squibb, AOP Orphan Pharmaceuticals and Cure Leukaemia. Bristol Myers Squibb are providing free fedratinib (only for the duration of the study) and they have provided a grant to cover the some of the costs of running the study. AOP Orphan Pharmaceuticals are providing Ropeginterferon alfa-2b for the duration of the study only and a grant to cover the some of the costs of running the study. The study is being run by the Haematology Team at the Cancer Research UK Clinical Trials Unit at the University of Birmingham.

8 Who has reviewed the study?

This research study has received independent peer review via the Cancer Research UK Clinical Trials Unit and also by an independent Research Ethics Committee. Research Ethics Committees review all research to protect the safety, rights, wellbeing, and dignity of participants. This study was reviewed and received favourable opinion by the Leeds West Ethics Committee. It has been reviewed and received a Clinical Trial Authorisation by the UK Competent Authority (Medicines and Healthcare products Regulatory Agency - MHRA) It has also been reviewed by the National Health Research Authority.

9 How have patients been involved in this study?

Patients who have lived experience of bone marrow cancer and its treatment have reviewed the protocol for this study as well as the patient documents. They have reviewed this information sheet and helped us to develop it so that it is clear and correct. They will also be involved in the ongoing management of this study, in order to make sure that the interests of patients are maintained.

10 Further information and contact details

If you have any questions or concerns about your disease or this research study, please discuss them with your doctor. You may also find it helpful to contact the following organisations:

<< insert name and contact telephone number of Principal Investigator >>

<< insert name and contact telephone number of Research Nurse >>

<< insert 24 hour emergency contact details >>

<<Delete as appropriate for your site>>

You may also find it helpful to contact the following organisations:

England

Your local Patient Advice and Liaison Service (PALs) or equivalent who provide advice and support to patients, their families and their carers, website:
http://www.nhs.uk/chq/Pages/1082.aspx?CategoryID=68&SubCategoryID=153
Or local PALS details where available:
<< insert address and contact telephone number of local service >>

Northern Ireland

In Northern Ireland the Patient Client Council (PCC) can provide assistance and support at any stage of the health and social care services complaints procedure. The PCC is an independent body who represent the views of the public in all areas of health and social care. They can also assist you to make a complaint. This is a confidential and free service.
http://www.patientclientcouncil.hscni.net/
Telephone: 0800 917 0222
Email: info.pcc@hscni.net

Scotland

The Patient Advice and Support Service is an independent service which provides free, accessible and confidential information, advice and support to patients, their carers, and families about NHS healthcare in Scotland.
http://www.patientadvicescotland.org.uk/

Wales

Community Health Councils (CHCs) are independent bodies, set up by law, who listen to what individuals and the community have to say about the health services with regard to quality, quantity, access to and appropriateness of the services provided for them. CHCs can also help, advise and support people who wish to make complaints about NHS services and similar matters. This advice is completely free, independent and confidential.
http://www.wales.nhs.uk/sitesplus/899/home

Sources of information

CancerHelp: an information service about cancer from Cancer Research UK, Freephone 0808 800 40 40, www.cancerhelp.org.uk

Macmillan Cancer Support: Freephone 0808 800 0000, www.macmillan.org.uk

CRUK Clinical Trial Database: https://www.cancerresearchuk.org/about-cancer/find-a-clinical-trial

Emergencies

If a medical emergency, related to your treatment for this study occurs while you are at home, you should initially try to contact the haematology unit where you received your treatment (see contact details below). If this is not possible you should go to the accident and emergency (A&E) department at your local hospital. If you are unable to get to the hospital you should contact your GP who will already have been informed of your participation in the study.

Please take as much time as you need to make a decision and then let your doctor know what you have decided so that your treatment can be arranged.

Thank you for taking time to read this leaflet and considering taking part in this study. You may use this information sheet to make notes or write down any questions you may have.
