# Supplementary material for: Fedratinib combined with ropeginterferon alfa-2b in patients with myelofibrosis (FEDORA): study protocol for a multicentre, open-label, Bayesian phase II trial
Source: BMC Cancer. 2025 Jan 10;25:56. doi: 10.1186/s12885-024-13383-3 (PMC11720754; doi:10.1186/s12885-024-13383-3)
Supplement: Supplementary file 4 — Supplementary Material 4: Appendix 4: FEDORA trial informed consent form. Exemplar informed consent form for the FEDORA trial. [file 12885_2024_13383_MOESM4_ESM.pdf]

Appendix 4: FEDORA trial informed consent form

Print on hospital headed paper

FEDORA

FEDORA: A phase II study to evaluate the tolerability, safety and activity of fedratinib combined with ropeginterferon alfa-2b in patients with myelofibrosis

INFORMED CONSENT FORM

**Site:**	**Patient Trial No. (TNO):**
**Principal Investigator:**	**EudraCT No:** 2021-004056-42

If you agree to take part in the FEDORA study, please:

- Initial each box
- Sign your full name at the end of this form

A. Taking part

1. I confirm that I have read and understand the FEDORA Patient Information Sheet, version_________, date________________________. I have had the opportunity to consider the information, ask questions and have had these answered to my satisfaction.

2. I understand that my participation is voluntary and that I am free to withdraw at any time without giving any reason, without my medical care or legal rights being affected.

   I understand that if I withdraw, some research may have already taken place using my samples and my data, and that this research cannot be undone.

3. I give permission for my date of birth and initials to be given to the Trial Office when I enter the FEDORA study, as well as a copy of this consent form to be sent to the Trial Office.

4. I agree to my GP being informed of my participation in this study.

5. I agree to take part in the FEDORA study.

B. Samples

6. I agree to donate as part of my participation in the FEDORA study:
   - Samples of my blood and serum
   - Samples of my bone marrow

7. I understand that my samples will be used for collecting DNA, genetic and additional analyses as described in the Patient Information Sheet.

8. I agree to allow my samples to be supplied to the Weatherall Institute of Molecular Medicine (WIMM) Laboratory at the University of Oxford, or other Human Tissue Act licensed facility or Research Organisation (as outlined in the Patient information Sheet) for analysis in relation to the FEDORA study. I understand that this FEDORA-specific analysis may take place at WIMM or other research organisation. I give permission for a copy of my consent form to be sent with the initial samples to the WIMM.

9. I understand that results and data from sequencing of my DNA will not be returned to me and may not help with my medical care now or in future

C. Data

10. I understand that relevant sections of my medical notes and data collected during the study may be looked at by individuals from the Trial Office, regulatory authorities, Sponsors, research collaborators, and/or NHS bodies, where it is relevant to my taking part in this research, safety monitoring, or licencing purposes. I give permission for these individuals to have access to my records.

11. I understand that my data, and information from my samples will only be used by researchers in a form that protects my anonymity. Anonymised data and information derived from research samples may be shared with other research organisations in future research. This may include academic institutions, clinical research groups or commercial (for-profit) companies. I understand that this data may be transmitted outside the European Economic area to countries which may have a different level of data protection to that in the UK.

Optional – *the following are optional and will not affect entry into the FEDORA study. Please initial for no or yes in the boxes:*

My samples or DNA from my samples could be shared with any UK research organisation that has research ethics committee approval or research organisations overseas that has country-specific approvals

	**No**	**Yes**
I agree to the storage of my samples remaining after analysis as part of this study, and for their use, and the use of my clinical data, in all other ethically approved research. This may involve research using animals and I agree to this.	☐	☐
I agree to the storage of my samples remaining after analysis as part of this study, and for their use, and the use of my clinical data, in other ethically approved research that does not involve animals.	☐	☐
I agree to complete the Quality of Life questionnaires at the time points specified in the Patient Information Sheet.	☐	☐

______________________________	______________	_________________________________
**Name of participant**	**Date**	**Signature**

______________________________	______________	_________________________________
**Name of person taking consent**<br>(You must have signed the Site Signature and Delegation Log)	**Date**	**Signature**

When completed, 1 for patient; 1 (original) for Investigator Site File; 1 to be kept in medical notes; 1 to be sent to CRCTU
