# Supplementary material for: Fedratinib combined with ropeginterferon alfa-2b in patients with myelofibrosis (FEDORA): study protocol for a multicentre, open-label, Bayesian phase II trial
Source: BMC Cancer. 2025 Jan 10;25:56. doi: 10.1186/s12885-024-13383-3 (PMC11720754; doi:10.1186/s12885-024-13383-3)
Supplement: Supplementary file 5 — Supplementary Material 5: Appendix 5: Dose modifications for fedratinib during FEDORA. The haematological and non-haematological toxicity guidance, as well as management of thiamine levels and Wernicke’s encephalopathy for use during FEDORA. [file 12885_2024_13383_MOESM5_ESM.docx]

# Appendix 5: Dose modifications for fedratinib during FEDORA

| **Toxicity observed** | **Dose reduction** |
| --- | --- |
| **Haematological toxicity** | |
| Grade 3 thrombocytopenia (<50 ×10^9^/L) with bleeding  Grade 4 thrombocytopenia (<25 ×10^9^/L) | Interrupt fedratinib until platelet count ≥75 ×10^9^/L. Restart at 100mg daily below the last given dose. |
| Grade 4 neutropenia (<0.5 ×10^9^/L) | Interrupt fedratinib until neutrophil count ≥1.5 ×10^9^/L, or baseline. Restart at 100mg daily below the last given dose.  Granulocyte growth factors may be used at investigator’s discretion. |
| Grade 3-4 anaemia (haemoglobin <80g/L), transfusion indicated | Interrupt fedratinib until haemoglobin concentration ≥100g/L, or baseline. Restart at 100mg daily below the last given dose. |
| Recurrent grade 4 haematological toxicity | Discontinuation, as per investigator’s discretion |
| **Non-haematological toxicity** | |
| Grade 3-4 nausea, vomiting or diarrhoea not responding to supportive measures within 48 hours | Interrupt fedratinib until resolved to ≤Grade 1 or baseline. Check for thiamine deficiency associated with gastrointestinal toxicity. Restart at 100mg daily below the last given dose. |
| Grade 3-4 rise in alanine transferase (ALT), aspartate aminotransferase (AST), bilirubin, amylase, or lipase concentration | Interrupt fedratinib until resolved to ≤Grade 1 or baseline. Restart at 100mg daily below the last given dose.  Monitor ALT, AST, bilirubin (total and direct), amylase and lipase every 2 weeks for at least 3 months following the dose reduction. If re-occurrence of grade 3-4 rise, discontinue treatment. |
| Grade 3-4 other non-haematological toxicity | Interrupt fedratinib until resolved to ≤Grade 1 or baseline. Restart at 100mg daily below the last given dose. |
| **Management of thiamine levels and Wernicke’s encephalopathy (WE)** | |
| For thiamine concentration below the lower limit of normal (LLN) but ≥30nmol/L, and no signs or symptoms of WE | Interrupt fedratinib treatment. Dose with thiamine 100mg orally two to three times daily until thiamine levels are restored to the normal range. Consider re-starting fedratinib when thiamine levels normal. |
| For thiamine concentration <30nmol/L, and no signs or symptoms of WE | Interrupt fedratinib treatment. Dose with thiamine therapeutic dosage parenterally until thiamine levels are restored to the normal range. Consider re-starting fedratinib when thiamine levels normal. |
| For signs or symptoms of WE, regardless of thiamine concentration | Discontinue fedratinib, and immediately administer parenteral thiamine at therapeutic dosage. |
