# Supplementary material for: Fedratinib combined with ropeginterferon alfa-2b in patients with myelofibrosis (FEDORA): study protocol for a multicentre, open-label, Bayesian phase II trial
Source: BMC Cancer. 2025 Jan 10;25:56. doi: 10.1186/s12885-024-13383-3 (PMC11720754; doi:10.1186/s12885-024-13383-3)
Supplement: Supplementary file 6 — Supplementary Material 6: Appendix 6: Concomitant medications to avoid or use with caution during FEDORA. Drugs known or expected to interact with ropeginterferon alfa-2b or fedratinib. [file 12885_2024_13383_MOESM6_ESM.docx]

Appendix 6 – Concomitant medications to avoid or use with caution

Drugs known or expected to interact with ropeginterferon alfa-2b or fedratinib are provided below. This list is not exhaustive, and the recommendations should be considered when co-administering other medications which interact with the same pharmacokinetic or pharmacodynamic processes.

A COVID-19 vaccine given to a trial participant is considered to be a simple concomitant medication with no interaction that requires advice on timing of the vaccine or other aspects.

Ropeginterferon alfa-2b

| **Drug group** | **Drug** | **Recommendation** |
| --- | --- | --- |
|  | Telbivudine | Contra-indicated |
| CYP1A2 substrates with narrow therapeutic index | Methadone, theophylline | Caution – ropeginterferon alfa-2b may increase exposure |
| CYP2D6 substrates | Atomoxetine, desipramine, dextromethorphan, eliglustat, nebivolol, nortriptyline, perphenazine, risperidone, tolterodine, R-venlafaxine | Caution – ropeginterferon alfa-2b may increase exposure |
| Myelosuppressive drugs | | Caution – ropeginterferon alfa-2b may enhance effect on bone marrow |
| Narcotics, hypnotics, sedatives | | Caution – ropeginterferon alfa-2b may enhance sedation |

Fedratinib

| **Drug group** | **Drug** | **Recommendation** |
| --- | --- | --- |
| Strong CYP3A4 inhibitors | Boceprevir, clarithromycin, cobicistat, elvitegravir, idelalisib, indinavir, itraconazole, ketoconazole, lopinavir, nefazodone, nelfinavir, posaconazole, rifampicin, ritonavir, saquinavir, telaprevir, telfinavir, telithromycin, troleandomycin, voriconazole  Grapefruit juice, Seville oranges | Avoid – increases exposure to fedratinib.  If use cannot be avoided, reduce fedratinib dose and monitor carefully (at least weekly) for safety. |
| Moderate CYP3A4 inhibitors | Aprepitant, ciclosporin, ciprofloxacin, conivaptan, crizotinib, diltiazem, dronedarone, erythromycin, fluconazole, imatinib, tofisopam, verapamil | Avoid prolonged administration – may increase exposure to fedratinib.  If use cannot be avoided, monitor carefully for safety, and modify dose according to adverse reactions. |
| CYP2C19 inhibitors | Fluoxetine, ticlopidine | Avoid – may increase exposure to fedratinib.  If use cannot be avoided, monitor carefully for safety, and modify dose according to adverse reactions. |
| Dual inhibitors of CYP3A4 and CYP2C19 | Fluconazole, fluvoxamine  (or combinations of the drugs described in the relevant sections above) | Avoid – may increase exposure to fedratinib. |
| Strong or moderate CYP3A4 inducers | Apalutamide, carbamazepine, efavirenz, enzalutamide, mitotane, phenytoin, rifampin, St. John’s wort  Bosentan, efavirenz, etravirine, phenobarbital, primidone | Avoid – may reduce exposure to fedratinib. |
| CYP3A4 substrates | Alfentanil, avanafil, buspirone, conivaptan, darifenacin, darunavir, ebastine, everolimus, ibrutinib, lomitapide, lovastatin, midazolam, naloxegol, nisoldipine, saquinavir, simvastatin, sirolimus, tacrolimus, tipranavir, triazolam, vardenafil | Caution – fedratinib may increase exposure. Monitor closely for safety and efficacy and modify doses of these drugs as required. |
| CYP2C19 substrates | S-mephenytoin, omeprazole |  |
| CYP2D6 substrates | Atomoxetine, desipramine, dextromethorphan, eliglustat, nebivolol, nortriptyline, perphenazine, tolterodine, R-venlafaxine |  |
