# Supplementary material for: Fedratinib combined with ropeginterferon alfa-2b in patients with myelofibrosis (FEDORA): study protocol for a multicentre, open-label, Bayesian phase II trial
Source: BMC Cancer. 2025 Jan 10;25:56. doi: 10.1186/s12885-024-13383-3 (PMC11720754; doi:10.1186/s12885-024-13383-3)
Supplement: Supplementary file 7 — Supplementary Material 7: Appendix 7: FEDORA Bayesian probability plots and operating characteristics. Posterior probability plots and operating characteristics for a range of possible scenarios within the FEDORA trial. [file 12885_2024_13383_MOESM7_ESM.pdf]

Appendix 7: FEDORA Bayesian probability plots and operating characteristics

Within this appendix, posterior probability plots have been presented using the binary outcome of tolerability; an uninformative Beta(1,1) prior is used throughout. These plots have been produced using an in-house written function in R.

For 30 Evaluable Patients

1) 18 patients tolerate treatment (60% observed tolerability)

We would be 11% sure that the tolerability rate was greater than 70% if 18 patients tolerated treatment. The plot below shows the distribution of tolerability and the red lines represent the 60%, 70% and 80% marks.

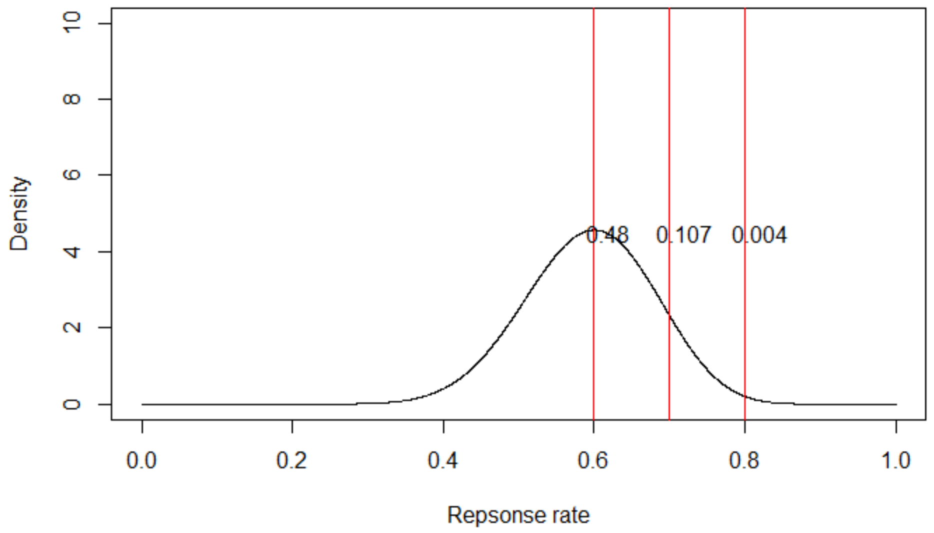

P (true tolerability rate > 60%) =	48%
P (true tolerability rate > 70%) =	11%
P (true tolerability rate > 80%) =	0%

2) 21 patients tolerate treatment (70% observed tolerability)

We would be 46% sure that the tolerability rate was greater than 70% if 21 patients tolerated treatment. The plot below shows the distribution of tolerability and the red lines represent the 60%, 70% and 80% marks

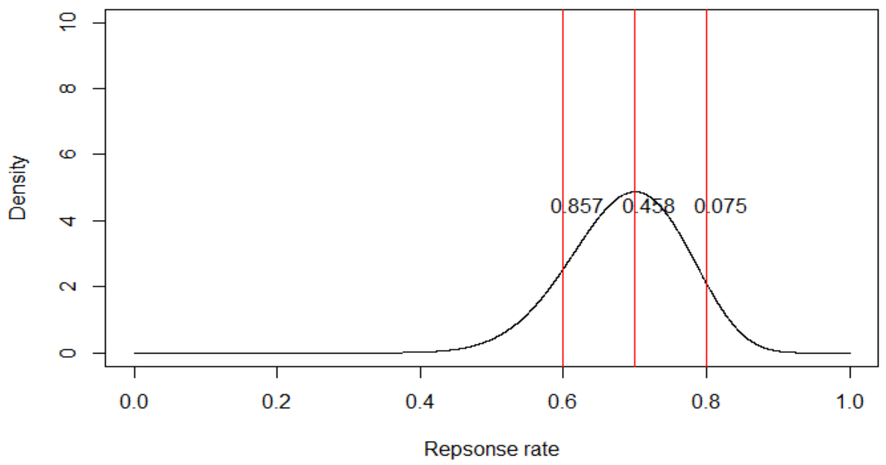

P (true tolerability rate > 60%) =	86%
P (true tolerability rate > 70%) =	46%
P (true tolerability rate > 80%) =	8%

3) 23 patients tolerate treatment (76% overserved tolerability)

We would be 76% sure that the tolerability rate was greater than 70% if 23 patients tolerated treatment. The plot below shows the distribution of tolerability and the red lines represent the 60%, 70% and 80% mark

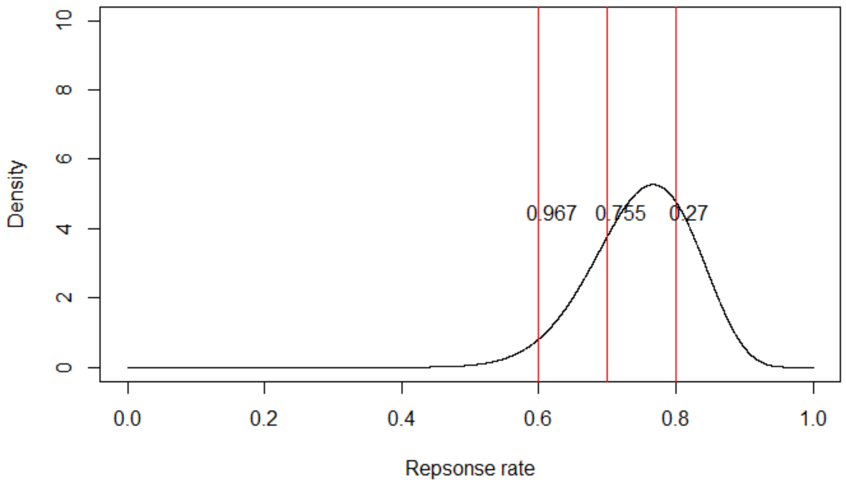

P (true tolerability rate > 60%) =	97%
P (true tolerability rate > 70%) =	76%
P (true tolerability rate > 80%) =	27%

4) 24 patients tolerate treatment (80% overserved tolerability)

We would be 87% sure that the tolerability rate was greater than 70% if 24 patients tolerated treatment. The plot below shows the distribution of tolerability and the red lines represent the 60%, 70% and 80% mark

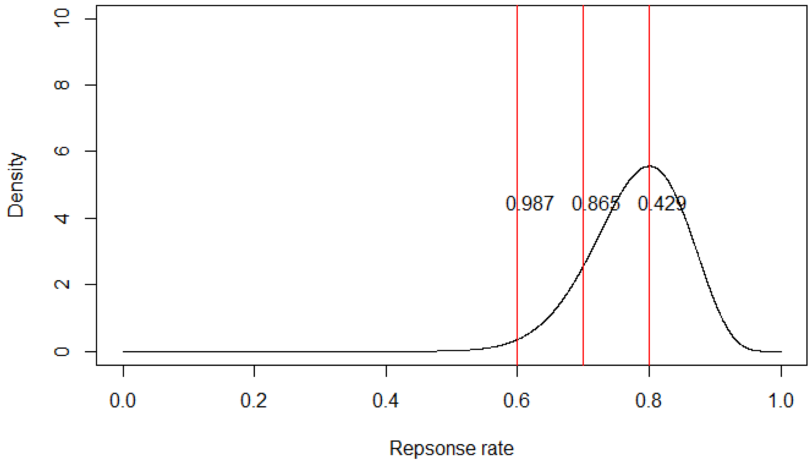

P (true tolerability rate > 60%) =	99%
P (true tolerability rate > 70%) =	87%
P (true tolerability rate > 80%) =	43%

In order to calculate operating characteristics for the FEDORA trial several simulations were conducted. This trial is a single arm trial with a binary outcome measure of tolerability, the simulations presented below replicate this trial design utilising a conjugate beta-binomial analysis 10,000 simulations, an uninformative Beta(1,1) prior, target rate of 70%, critical rate of 70% and 30 patients. Simulations were conducted for a range of true underlying tolerability rates; 50%, 60%, 70%, 75% and 80%, the results of each of these simulations are presented below. The acceptance rate presented represents the proportion of times in each scenario that we conclude the true tolerability rate in each scenario to be greater than the target rate.

From the below table, in the scenario, (Scenario 1), where the true tolerability rate is lower than both the target and the critical rate the produced acceptance rate is less than 1% indicating that there is a very low chance that we will conclude incorrectly that the true tolerability rate is greater than 70%. In the scenario where the true tolerability rate is above the target rate by 10%, (Scenario 5), the produced acceptance rate, the proportion of times we make the correct conclusion, is greater than 70%. This indicates that there is a low chance that we will make the incorrect conclusion if we observe a true tolerability rate greater than the targeted 70%.

Scenario	Number of Simulations	Number of Patients	Critical Rate	Target Rate	True Tolerability Rate	Acceptance Rate
1	10,000	30	70%	70%	50%	0.24%
2					60%	4.13%
3					70%	28.1%
4					75%	52.8%
5					80%	76.0%
